# The Need to Analyze Telogen Effluvium and Alopecia Areata Parallelly in Long COVID Studies

**DOI:** 10.1111/irv.13356

**Published:** 2024-07-26

**Authors:** Chia‐Tse Weng, Kai‐Che Wei, Chao‐Chun Yang

**Affiliations:** ^1^ Division of Allergy, Immunology and Rheumatology, Department of Internal Medicine, National Cheng Kung University Hospital, College of Medicine National Cheng Kung University Tainan Taiwan; ^2^ Department of Dermatology Kaohsiung Veterans General Hospital Kaohsiung Taiwan; ^3^ College of Medicine National Yang Ming Chiao Tung University Taipei Taiwan; ^4^ Department of Dermatology, National Cheng Kung University Hospital, College of Medicine National Cheng Kung University Tainan Taiwan

**Keywords:** alopecia areata, telogen effluvium, long COVID

## Peer Review

The peer review history for this article is available at https://www.webofscience.com/api/gateway/wos/peer‐review/10.1111/irv.13356.

Dear Editor,

I am writing in response to the article titled “Risks of alopecia areata in long COVID: Binational population‐based cohort studies from South Korea and Japan” by Kyung et al., recently published in the Journal of Medical Virology [1]. The study provides robust evidence on the association between SARS‐CoV‐2 infection and the increased risk of developing alopecia areata (AA) as part of long COVID. It also highlights the significant impact of COVID‐19 severity and vaccination status on AA risk.

Telogen effluvium (TE) is another type of hair loss that could be highly relevant to COVID‐19 and long COVID [2]. TE and AA have overlapping clinical manifestations, but TE is not as well‐known as AA by physicians. Therefore, misclassification between TE and AA is possible. TE is characterized by diffuse hair shedding often triggered by significant stress, illness, or hormonal changes [3]. Given the profound stress and physiological changes associated with COVID‐19, TE is a common postinfection manifestation [4]. Furthermore, the severity of COVID‐19 has been correlated with an increased risk of TE [5].

To provide a comprehensive understanding of post‐COVID‐19 hair loss patterns, it is advisable to present TE and AA in parallel using the existing database. This approach could yield significant insights into the prevalence of hair loss in long COVID. While the database may not confirm the accuracy of AA versus TE diagnoses, presenting results for both conditions can help clarify their respective impacts.

It is prudent to acknowledge that dermatologists have a relatively clear understanding of the differences between TE and AA. Therefore, it might be beneficial for the authors to consider limiting AA diagnoses to those confirmed by dermatologists to enhance diagnostic accuracy and reliability. Furthermore, comparing hair loss caused by other viral infections, such as influenza, which is more frequently reported to cause AA and less often reported to cause TE [6], can enhance the overall understanding of virus‐associated alopecia.

In conclusion, while the study by Kyung et al. provides significant insights into the risk of AA following COVID‐19, incorporating the diagnosis of TE, applying stricter criteria for diagnosing AA, and considering additional control groups in future research would offer a more holistic view of postinfection hair loss. This approach could enhance our understanding of long COVID and improve patient care strategies.

## Author Contributions

Chia‐Tse Weng and Kai‐Che Wei wrote the manuscript. Chao‐Chun Yang substantively revised it. All authors read and approved the final manuscript.

## Conflicts of Interest

The authors declare no conflicts of interest.

## Data Availability

The authors have nothing to report.
